# Role of 4-aminobutyrate aminotransferase (ABAT) and the lncRNA co-expression network in the development of myelodysplastic syndrome

**DOI:** 10.3892/or.2022.8447

**Published:** 2022-11-16

**Authors:** Yanzhen Chen, Guangjie Zhao, Nianyi Li, Zhongguang Luo, Xiaoqin Wang, Jingwen Gu

Oncol Rep 42: 509–520, 2019; DOI: 10.3892/or.2019.7175

Subsequently to the publication of the above article, and a Corrigendum that has already been published with the intention of showing corrected versions of Figs. 1 and 8 (DOI: 10.3892/or.2022.8348; published online on June 14, 2022), the authors have belatedly realized that the revisions made to Fig. 8 necessitated changes that should have been introduced into Fig. 9, although these were not attended to in the first corrigendum. Essentially, Fig. 8 was revised as the cell apoptosis and cell proliferation assays therein were poorly presented, which made the interpretation of the data difficult; Fig. 9 showed the fractions of apoptotic cells in the SKM-1 and THP-1 cell lines with lncENST00000444102 overexpression as this pertained to Fig. 8.

A revised version of Fig. 9, presenting the analysis of the data shown in the revised version of Fig. 8, is shown opposite. In addition to the revision of Fig. 9, the sentence starting on p. 517, left-hand column, line 12 [“The flow cytometric apoptosis assay revealed that lncENST00000444102 overexpression promoted tumor cells to undergo apoptosis compared to control cells (P<0.001, Fig. 9)”] should be replaced with the following text, to reflect the change in the level of statistical significance: ‘The flow cytometric apoptosis assay revealed that lncENST00000444102 overexpression promoted tumor cells to undergo apoptosis compared to control cells (**P<0.01**, Fig. 9)”.

Note that the revisions made to Figs. 8 and 9 in this paper have not had a major impact on the reported results, and do not affect the overall conclusions reported in the study. All the authors agree to the publication of this corrigendum. The authors are grateful to the Editor of *Oncology Reports* for allowing them the opportunity to publish this additional Corrigendum; furthermore, they apologize for any inconvenience caused to the readership of the Journal.

## Figures and Tables

**Figure 9.**
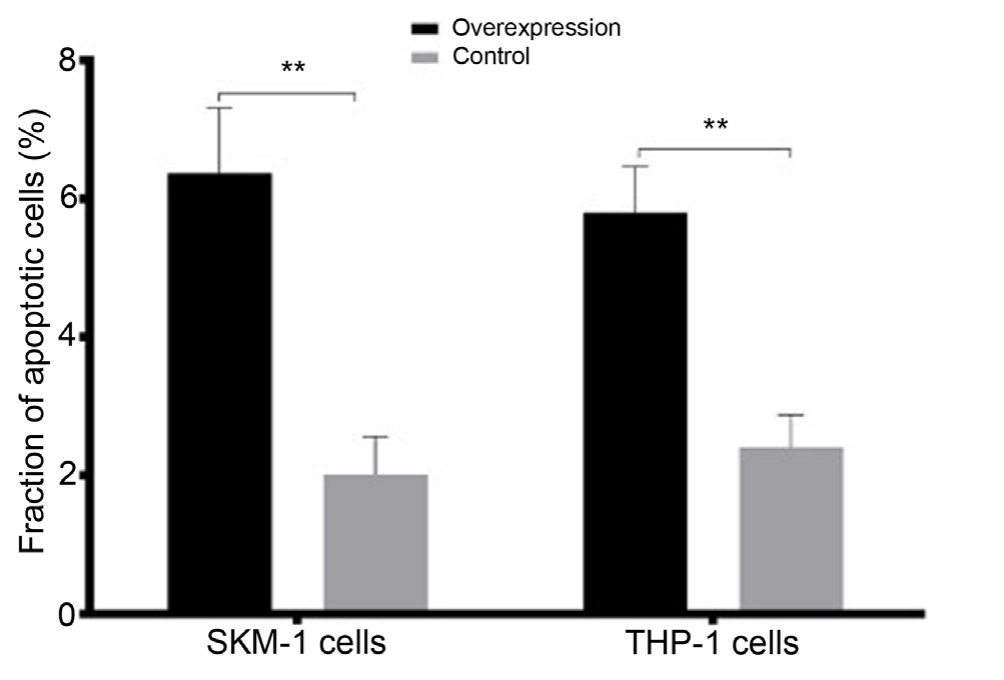
The fraction of apoptotic cells in the SKM-1 and THP-1 cell lines with lncENST00000444102 overexpression. Data are presented as the means ± standard deviation. ^**^P<0.01 vs. the controls.

